# Primary retroperitoneal extraskeletal mesenchymal chondrosarcoma involving the vena cava: A case report

**DOI:** 10.3892/ol.2014.2012

**Published:** 2014-03-28

**Authors:** HUI-JUAN HU, MEI-YAN LIAO, LI-YING XU

**Affiliations:** Department of Computed Tomography, Zhongnan Hospital of Wuhan University, Wuhan, Hubei 430071, P.R. China

**Keywords:** extraskeletal chondrosarcoma, mesenchymal, X-ray computed tomography, magnetic resonance imaging

## Abstract

The current study presents a case of extraskeletal mesenchymal chondrosarcoma (ESMC) involving the vena cava that originally occurred in the retroperitoneum of a 61-year-old female. Following excision of the masses, pathological examination confirmed a diagnosis of primary ESMC. Mesenchymal chondrosarcomas are extremely rare in comparison to conventional chondrosarcomas and even more so when arising in an extraskeletal location. In the current report, the major characteristics of ESMC are discussed and a review of the current knowledge regarding this rare disease entity is presented.

## Introduction

Mesenchymal chondrosarcoma is a rare and aggressive variant subtype of chondrosarcoma, which represents ~1% of all chondrosarcomas. It commonly arises in the bone, however, in ~30–40% of cases, it occurs in an extraskeletal location (1).

Extraskeletal mesenchymal chondrosarcoma (ESMC) may occur in any location that contains mesenchymal cells, however, the majority arise in the lower extremities (particularly the thigh), leptomeninges or the orbit (2). Other sites, including the retroperitoneum, are extremely rare and to the best of our knowledge, no cases of primary retroperitoneal ESMC involving the vena cava have previously been reported. The current study presents the case of a 61-year-old female with primary retroperitoneal ESMC involving the vena cava. Patient provided written informed consent.

## Case report

On 27th June 2013, a 61-year-old female presented to Zhongnan Hospital of Wuhan University (Wuhan, China) with a six-month history of persistent abdominal pain and distension, occasional reflux and nausea, as well as depression, fatigue and weight loss of 10 kg. The patient had no history of any fever, night sweats, chest pain, diarrhea or blood loss in the stools. The patient’s general physical and chest examinations were unremarkable, however, the abdominal examination revealed right subabdominal tenderness and multiple hard lumps of different sizes. The patient’s past medical and personal histories included cardiac disease, hysteromyoma and peritoneal myxoma of 12 years. The laboratory results revealed the patient’s hemoglobin levels to be 80 g/l with a red cell count of 2.61×10^12^ cells/l. In addition, the patient’s serum lactate dehydrogenase levels were 487 U/l (normal range, 135–225 U/l) and mannosylated antigen CA125 levels were 206.5 U/ml (normal range, 0–35 U/ml).

**Figure 1:**
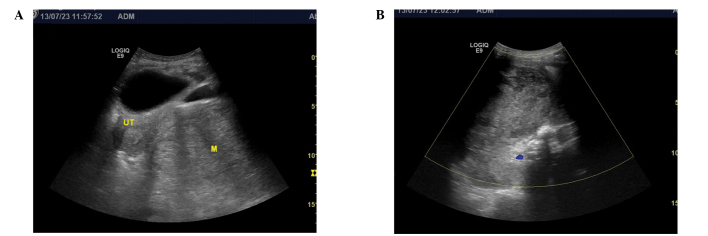
Abdominal and pelvic ultrasound. (A and B) Solid heterogeneous retroperitoneal masses are evident, with multiple scattered areas of increased echogenicity and dense posterior shadowing suggestive of foci of calcification.

Abdominal and pelvic ultrasound examinations were performed and revealed two huge, heterogeneous retroperitoneal masses located on the rectouterine fossa adjacent to the inferior vena cava, which measured between 126×103 and 95×78 mm in size (Fig. 1A and B). The tumors exhibited multiple scattered areas of increased echogenicity with dense posterior shadowing that suggested foci of calcification.

**Figure 2:**
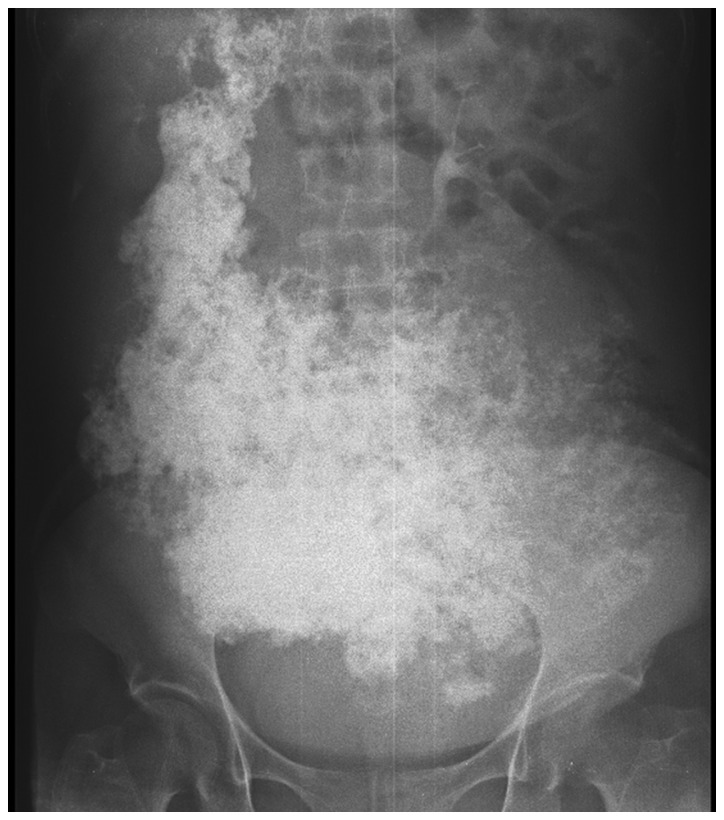
Plain radiography showing extensive calcifications in the abdomen.

**Figure 3:**
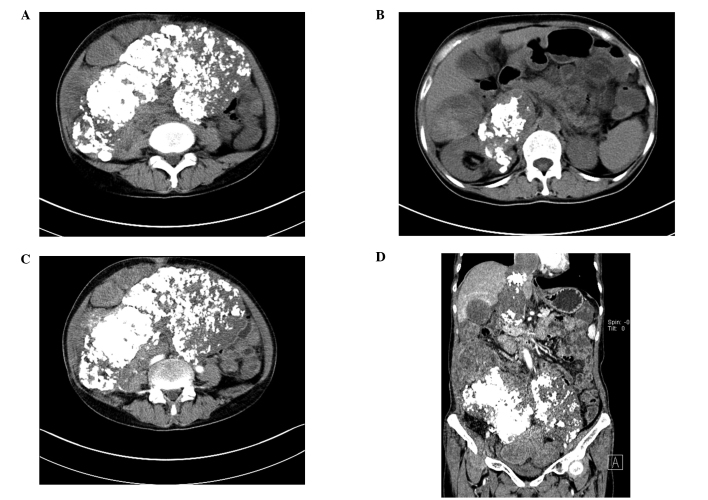
(A and B) Unenhanced transverse CT reveals heterogeneous masses with extensive dense, as well as ring- and arc-like calcifications in the retroperitoneum and inferior vena cava. (C) Enhanced transverse CT reveals subtle heterogenous enhancement in the periphery of the mass, but no enhancement is evident in the majority of the mass. (D) Enhanced coronal CT shows a clear mass in the inferior vena cava. CT, computed tomography.

The plain radiography revealed extensive calcifications in the abdomen (Fig. 2). Furthermore, unenhanced transverse computed tomography (CT) scans of the abdomen and pelvis revealed multiple different sized masses located in the right hepatorenal recess, retroperitoneum (Fig. 3A), inferior vena cava (Fig. 3B) and rectouterine fossa. The masses appeared as heterogeneous masses with extensive and dense, as well as ring- and arc-like calcifications. Contrast-enhanced CT was performed following the mechanical injection of 80 ml non-ionic iopromide (370 mg/ml of iodine) into the antecubital vein at a rate of 3.0 ml/sec. Following the initiation of the infusion of contrast material, images were captured after a scanning delay of 45 sec. The contrast-enhanced transverse CT scan revealed subtle heterogenous enhancement in the periphery of the masses, however, the majority of masses were not found to demonstrate enhancement (Fig. 3C). In addition, the mass in the inferior vena cava was clearly identifiable in the coronal plane (Fig. 3D).

**Figure 4:**
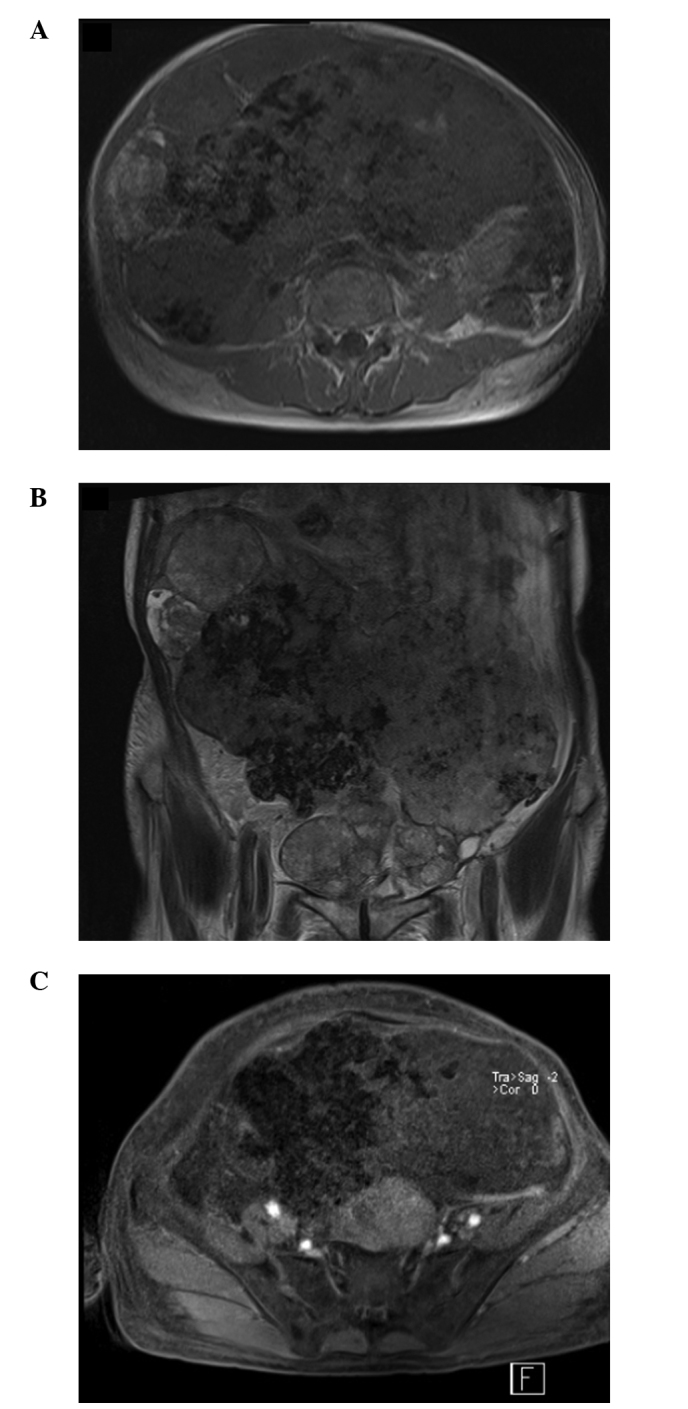
(A) Unenhanced transverse T1-weighted images reveal low signal intensity of the lesions, whereas the central area exhibits an even lower intensity. (B) Unenhanced coronal T2-weighted images predominantly reveal marginally high signal intensities of the lesions, whereas the central area exhibits low intensity. (C) Enhanced magnetic resonance imaging reveals peripheral and mild speculated enhancement.

The patient also underwent abdominal and pelvic magnetic resonance imaging (MRI) (Siemens Trio 3.0T; Siemens Medical Solutions USA, Inc., Malvern, PA, USA) examinations and corresponding gadolinium-based contrast enhancement. On T1-weighted images (T1W1), all lesions showed low signal intensity and on T2-weighted images (T2WI), all lesions predominantly showed slightly high signal intensity, whereas the central area exhibited low intensity in the two images (Fig. 4A and B). Furthermore, enhanced MRI revealed peripheral and mild speculated enhancement around the lesions, which was considered to be the result of calcification (Fig. 4C).

**Figure 5:**
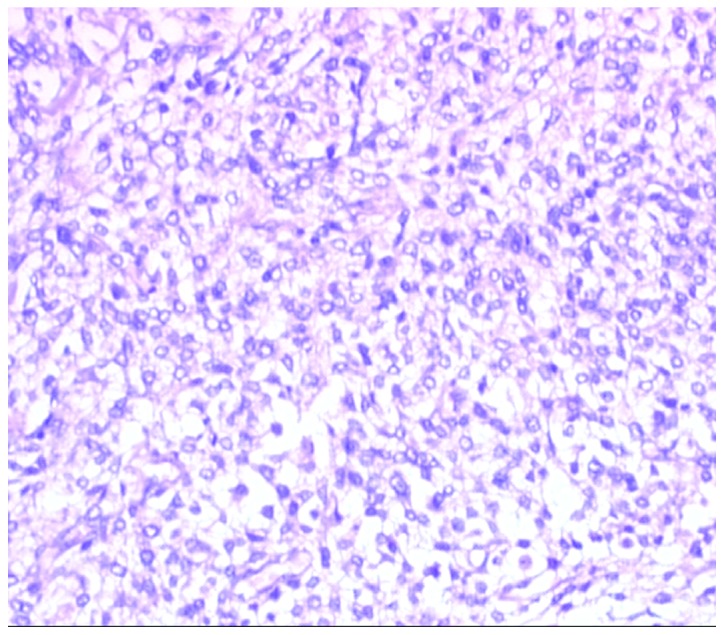
Photomicrograph of the surgical specimen revealing undifferentiated oval or spindle-shaped cells and small, benign-appearing cartilaginous tissue (stain, hematoxylin and eosin; magnification, ×100).

**Figure 6:**
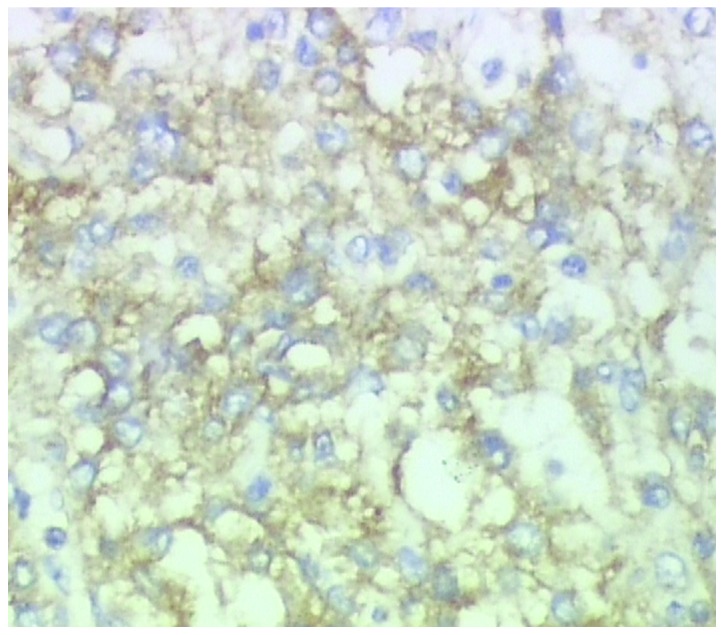
Immunohistochemical staining of the specimen revealing positive staining in the cells for CD99 (stain, CD99; magnification, ×400).

Subsequently, the patient underwent resection of the masses and the histological examination of the masses revealed observations consistent with those of ESMC (Fig. 5). In addition, immunohistochemistry of the cells showed reactivity for CD99 (Fig. 6), but was negative for CD117, Dog1, CD34, S100, CD31, myogenic differentiation 1, CD138, CD1a, CD20, CD3, synaptophysin, chromogranin A and Bcl-6. Additionally, the Ki-67 index was 60%.

## Discussion

Chondrosarcomas are a heterogeneous group of malignant neoplasms that produce a cartilaginous matrix. Although the majority of chondrosarcomas arise from cartilaginous or bony structures, they may also develop in extraskeletal locations, such as the soft tissues, where cartilage is not usually found. The histological subtypes of extraskeletal chondrosarcoma include myxoid, mesenchymal and well-differentiated, with the myxoid subtype being the most common (3). This highly malignant, cartilage-producing sarcoma was first described in the bone by Lichtenstein and Bernstein in 1959 (4), and in the soft tissue by Dowling in 1964 (5). Compared with the myxoid subtype, ESMC is rare, more aggressive and exhibits a poor prognosis (6). Furthermore, ESMC accounts for <1% of all sarcomas, which are located mainly in the orbit, the cranial and spinal meningeal coverings, the lower limbs and particularly in the thigh (2). In rare instances, this type of tumor has also been found to arise in the mediastinum, hand musculature, retroperitoneum and kidney (1). A literature search, limited to the English language, identified few cases of ESMC of the retroperitoneum and no reported cases of primary retroperitoneal ESMC involving the vena cava.

ESMC has a marginal female predominance and has two peak ages of incidence in adults, depending on its location; 23.5 years old (range, 5–48 years old) for EMSC patients with central nervous system involvement and 43.9 years old (range, 21–62 years old) for ESMC patients with soft-tissue and/or muscular involvement (7). The patient presented in the current study was a 61-year-old female.

Ultrasonography is not a conventional diagnostic tool for mesenchymal chondrosarcoma, however, it can be used as the first imaging modality when these masses arise from soft tissue. The images usually reveal a solid, heterogeneous mass, often containing scattered areas of increased echogenicity and posterior shadowing consistent with the features of multiple foci of calcification (8,9).

Conventional radiography has less impact on the diagnosis of ESMC due to a lower density resolution and the overlapping of adjacent structures. CT depicts ESMC as a soft tissue mass with ring- and arc-like, stippled and highly opaque calcifications. Contrast-enhanced CT depicts EMSC as a soft tissue mass, with a portion of enhancing viable tumor in the periphery and central focal low attenuation areas that possibly present necrosis (2). In the present case of EMSC, the CT scan revealed extensive and dense, as well as ring- and arc-like calcifications in the lobulated soft tissue mass. The ring-and-arc mineralization was the most important of the imaging observations associated with ESMC and, thus, is of important value in the qualitative diagnosis. Calcification has also been reported to be common (67%), but not extensive in mesenchymal chondrosarcoma (10), however, in the present study the patient exhibited wide and dense calcification.

MRI of ESMC patients often show equal or low signal intensity on T1W1 and mixed high and low signal intensity on T2WI. Since the intratumoral calcified and non-calcified components of ESMC demonstrate low and high intensity on T2WI, respectively, they are visible as areas of high signal intensity surrounding areas of low signal intensity or as the ‘salt and pepper’ sign. Such observations have previously been reported and are considered an important characteristic of the disease (11). In addition, a diffuse heterogeneous or nodular enhancement has been observed in calcified and non-calcified areas following enhanced scanning, which are important diagnostic signs for ESMC (11,12). In the present study, the patient showed similar CT and MRI observations.

However, the imaging results of the present study did not enable the differentiation of ESMC from other neoplasms. Thus, a definitive diagnosis was determined based on the histological features. Microscopically, the most common feature of mesenchymal chondrosarcoma is a biphasic pattern composed of sheets of undifferentiated round, oval or spindle-shaped cells, as well as small, usually well-defined, islets of well-differentiated, benign-appearing cartilaginous tissue (13). Immunohistochemistry may further aid the differentiation of these lesions from other mimicking lesions, as ESMC is positive for CD99 and vimentin in the mesenchymal cells and S-100 in the chondroid areas (14). S-100 protein was initially found in the majority of ESMCs, however, studies have shown that <20% of ESMCs are S-100-positive (15). In addition, CD99 positivity is observed in Ewing’s sarcoma and has also been reported as a potential positive marker for mesenchymal chondrosarcoma (7). Currently, no specific immunohistochemical markers have been determined for ESMC, however, the present case was positive for CD99 expression.

Surgery is usually the primary treatment modality for mesenchymal chondrosarcoma. However, if the tumor cannot be completely resected or in cases of recurrent lesions, radiation therapy and chemotherapy must be considered (14). Although, the benefits of chemotherapy and/or radiation therapy remain unclear. The prognosis of mesenchymal chondrosarcoma is poor due to the high probability of metastases, which often occur several years following the initial treatment. Furthermore, Nakashima *et al* (13) reported a five-year survival rate of 54% and a 10-year survival rate of 27%.

In conclusion, the current study presents the first case of retroperitoneal extraskeletal myxoid chondrosarcoma involving the vena cava. Although imaging features of ESMC are non-specific, we speculate the following characteristics: i) Calcification, particularly the ring- and arc-like calcifications; ii) ‘salt and pepper’ sign on T2WI; iii) diffuse heterogeneous or nodular enhancement; and iv) areas strengthened by calcification. Therefore, typical dense and arc-like calcifications may confirm the diagnosis of mesenchymal chondrosarcoma of extraskeletal origin.

## References

[1] González-Cámpora R, Otal Salaverri C, Gomez Pascual A, Hevia Vazquez A, Galera Davidson H (1995). Mesenchymal chondrosarcoma of the retroperitoneum. Report of a case diagnosed by fine needle aspiration biopsy with immunohistochemical, electron microscopic demonstration of S-100 protein in undifferentiated cells. Acta Cytol.

[2] Shapeero LG, Vanel D, Couanet D, Contesso G, Ackerman LV (1993). Extraskeletal mesenchymal chondrosarcoma. Radiology.

[3] Taori K, Patil P, Attarde V, Chandanshive S, Rangankar V, Rewatkar N (2007). Primary retroperitoneal extraskeletal mesenchymal chondrosarcoma: a computed tomography diagnosis. Br J Radiol.

[4] Lichtenstein L, Bernstein D (1959). Unusual benign and malignant chondroid tumors of bone. A survey of some mesenchymal cartilage tumors and malignant chondroblastic tumors, including a few multicentric ones, as well as many atypical benign chondroblastomas and chondromyxoid fibromas. Cancer.

[5] Dowling EA (1964). Mesenchymal chondrosarcoma. J Bone Joint Surg Am.

[6] Bertoni F, Picci P, Bacchini P (1983). Mesenchymal chondrosarcoma of bone and soft tissues. Cancer.

[7] Müller S, Söder S, Oliveira AM, Inwards CY, Aigner T (2005). Type II collagen as specific marker for mesenchymal chondrosarcomas compared to other small cell sarcomas of the skeleton. Mod Pathol.

[8] Lange TA, Austin CW, Seibert JJ, Angtuaco TL, Yandow DR (1987). Ultrasound imaging as a screening study for malignant soft tissue tumors. J Bone Joint Surg Am.

[9] Johnson DB, Breidahl W, Newman JS, Devaney K, Yahanda A (1997). Extraskeletal mesenchymal chondrosarcoma of the rectus sheath. Skeletal Radiol.

[10] Murphey MD, Walker EA, Wilson AJ, Kransdorf MJ, Temple HT, Grannon FH (2003). From the archives of the AFIP: imaging of primary chondrosarcoma: radiologic-pathologic correlation. Radiographics.

[11] Hashimoto N, Ueda T, Joyama S, Araki N, Beppu Y, Tatezaki S, Matsumoto S (2005). Extraskeletal mesenchymal chondrosarcoma: an imaging review of ten new patients. Skeletal Radiol.

[12] Yang BT, Wang ZC, Liu S, Xian JF, Zhang ZY, Liu ZL, Lan BS (2006). CT and MRI diagnosis of chondrosarcoma in sinonasal and orbital region. Zhonghua Fang She Xue Za Zhi.

[13] Nakashima Y, Unni KK, Shives TC, Swee RG, Dahlin DC (1986). Mesenchymal chondrosarcoma of bone and soft tissue: A review of 111 cases. Cancer.

[14] Yang BT, Wang YZ, Wang XY, Wang ZC (2012). Mesenchymal chondrosarcoma of the orbit: CT and MRI findings. Clin Radiol.

[15] Lucas DR, Fletcher CD, Adsay NV, Zalupski MM (1999). High-grade extraskeletal myxoid chondrosarcoma: a high-grade epithelioid malignancy. Histopathology.

